# Variation in outcomes of the Melbourne Infant, Feeding, Activity and Nutrition Trial (INFANT) according to maternal education and age 2 and 3·5 years post-intervention

**DOI:** 10.1017/S1368980021000045

**Published:** 2021-04

**Authors:** Christine Delisle Nyström, Adrian J Cameron, Karen J Campbell, Kylie D Hesketh

**Affiliations:** 1 Department of Biosciences and Nutrition, Karolinska Institutet, Neo, Huddinge 141 83, Sweden; 2 Institute for Physical Activity and Nutrition (IPAN), Deakin University, Geelong, Australia; 3 Global Obesity Centre, Institute for Health Transformation, Deakin University, Geelong, Australia

**Keywords:** Children, Long-term follow-up, Moderation, Nutrition, Physical activity

## Abstract

**Objective:**

This study aimed to assess whether the long-term effectiveness of the Melbourne Infant, Feeding, Activity and Nutrition Trial (INFANT) at 2 and 3·5 years post-intervention varied according to maternal education and age.

**Design:**

Two and 3·5 years post-intervention follow-up of the INFANT cluster-randomised controlled trial. Outcomes at both follow-ups included children’s BMI *z*-scores, physical activity (ActiGraph), television viewing (parental report) and dietary intake (3 × 24-h dietary recalls). Dichotomous moderator variables included maternal education (university *v*. no university) and age (< 32 *v*. ≥ 32 years).

**Setting:**

Population based.

**Participants:**

Families completing the 15-month programme (*n* 492) were invited to participate in the follow-ups when their child was 3·6 and 5 years old.

**Results:**

At the 2-year follow-up, the intervention effects on vegetable (positive) and sweet snack (negative) intake were greater in children with higher educated mothers, whereas water consumption (positive) was greater in children with lower educated mothers. At the 2-year follow-up, the intervention was more effective in increasing water consumption in children with younger mothers and decreasing sweet snack intake in children with older mothers (opposite result observed at the 3·5-year follow-up). At the 3·5-year follow-up, children with younger and older mothers increased and decreased their consumption of savoury snacks, respectively.

**Conclusions:**

Moderation by maternal education and age were observed for some outcomes; however, clear patterns were not evident at both follow-ups, with little consistency across outcomes. This indicates that INFANT was more-or-less equally effective in children irrespective of their mother’s education level or age, which is important in community-based interventions.

Childhood overweight and obesity are growing public health issues worldwide regardless of country income. Approximately 38 million children under 5 years are classified as overweight or obese globally^(1)^. More specifically, recent Australian data show the prevalence of overweight and obesity is 11 and 9 %, respectively, among children aged 2–4 years^(2)^. This is of concern as overweight and obesity track from childhood into adolescence, with a recent study finding that approximately 90 % of 3-year-olds with obesity were overweight or obese when they were between 15 and 18 years of age^(3)^. Furthermore, Geserick *et al.* ^(3)^ found that for adolescents with obesity, the fastest weight gain happened between the ages of 2 and 6. Reversal of overweight and obesity through interventions is difficult once established, demonstrating the urgent need to focus on primary prevention^(4)^.

The Melbourne Infant, Feeding, Activity and Nutrition Trial (INFANT) was a community-based cluster-randomised controlled trial (2008–2010) focusing on the prevention of obesity-related behaviours in first-time Australian parents with infants aged 4 months^(5)^. Post-intervention (15 months after baseline), children in the intervention group compared to the control group consumed significantly less sweet snacks and watched significantly less television^(6)^. At the long-term follow-ups at 2 and 3·5 years post-intervention, that is, when the children were 3·6 and 5 years old, sustained effects for some obesity-related behaviours were observed. More specifically, positive intervention effects were observed for fruit, vegetable, sweet snack and water intake at the 2-year follow-up and for non-core drinks and sweet snack intake at the 3·5-year follow-up. Additionally, at both time points television viewing was around 10 min less per day in children in the intervention group^(7)^.

Maternal education has been found to be a strong predictor of child BMI, with lower maternal education associated with higher BMI from as early as 3 years of age^(8)^. Furthermore, in a recent systematic review, it has been observed that higher maternal educational attainment and age (≥ 30 years) are related to healthier dietary patterns in infants^(9)^. As maternal education and age have been found to be important determinants in obesity-related behaviours in childhood, it is important to assess the variations in outcomes of interventions by these indicators. Following INFANT, it was found that maternal age and educational attainment moderated the intervention effects for some obesity-related behaviours in infants aged 20 months^(10)^, that is, additional positive impacts of the intervention were observed for these subgroups. More specifically, the intervention increased vegetable intake and decreased sweet snack consumption in infants whose mothers had a higher education, whereas the intervention significantly increased water consumption in mothers with a lower education. With regard to maternal age, it was found that the intervention increased the consumption of both vegetables and water in infants with younger mothers^(10)^.

There is strong support for long-term follow-up of intervention studies in order to examine sustained effects^(4,11)^. However, to date, few trials have investigated moderators in obesity prevention trials in young children and even fewer have explored moderators in long-term follow-ups. In the area of early childhood obesity prevention, Yavuz *et al.* ^(12)^ in their meta-analysis of fifty studies stated that more obesity prevention studies in early childhood need to perform long-term follow-up in order to better understand the moderators that are associated with sustained effects. Thus, the aim of this study is to assess the effectiveness of INFANT by maternal education and age at 2 and 3·5 years post-intervention.

## Methods

### Study design and participants

INFANT’s study design, sample selection, intervention and primary outcomes have been described previously^(5,6)^. Briefly, the control group received usual care from the maternal and child health nurse, whereas the intervention group received usual care plus 6 × 2-h group sessions delivered by a dietician over the 15-month intervention period^(5,6)^.

A total of 542 children were randomised into the study, with a total of 492 (91 %) completing the trial when the child was 20 months old. All 492 families who completed the programme post-intervention were contacted and asked to partake in the follow-ups when their child was 3·5 and 5 years old^(13)^. At the 2- and 3·5-year follow-ups, a total of 358 and 362 children, respectively, had outcome data for anthropometrics, physical activity, television viewing or diet.

### Measures

At both follow-ups, all measures for anthropometrics, physical activity, television viewing and dietary intake were identical.

#### Anthropometrics

Height and weight were assessed when the children were wearing light clothing using a portable stadiometer (Seca 220/217) and scale (Tanita BWB-800/InnerScan 50), respectively. Height and weight were assessed twice and recorded to the nearest 0·1 cm for height and 10 g for weight. The average of the two measurements was used to calculate BMI. BMI *z*-scores were then computed using the gender-specific BMI-for-age growth charts from the WHO^(14)^.

#### Physical activity

The children’s total physical activity (i.e., light, moderate and vigorous intensity) was assessed together using the ActiGraph accelerometer (Model GT1M). The children wore the accelerometers for eight consecutive days above their right hip and were instructed to only take the monitors off for water-based activities (e.g., showering or swimming) and sleeping. The ActiGraphs collected data using 15-s epochs and to be included in the analyses children had to have at least 4 d of valid data where the monitors were worn for a minimum of 7·4 h/d^(15)^. Within this sample, a minimum of 4 d has been found to provide an acceptable reliability estimate (interclass correlation coefficient > 0·70) for total physical activity^(15)^. The cut-points by Janssen *et al.* ^(16)^ were used to assess the amount of time spent in physical activity intensities.

#### Television viewing

The amount of time spent watching television was assessed in the parental questionnaire by the following two questions: ‘on an average weekday (Monday–Friday) how much time does your child spend watching or in front of the television?’ and ‘on an average weekend day (Saturday–Sunday) how much time does your child spend watching or in front of the television?’. These questions have been found to have acceptable reliability (interclass correlation coefficient 0·69, 95 % CI 0·54, 0·80) in a separate sample of Australian parents with a 3- to 5-year-old child^(17)^.

#### Dietary intake

Three multi-pass 24-h dietary recalls^(18)^ over the phone with a parent were used to assess the children’s diet and this procedure has been described previously^(6,10)^. The current analyses utilised the mean daily consumption (in g/d) of fruit (omitting fruit juice), vegetables (omitting potatoes), non-core sweet foods (e.g., bakery products, candy), non-core savoury foods (e.g., potato chips, savoury crackers), non-core drinks (e.g., soda, fruit juice), and water.

### Moderator variables

#### Maternal education

The highest obtained level of education was self-reported in the parental questionnaire when the families were first enrolled in the study when the infant was 4 months old. Maternal education was dichotomised into two groups for these analyses in order to maximise power (university degree *v*. no university degree).

#### Maternal age

The mother’s date of birth was self-reported in the parental questionnaire at baseline and maternal age was computed from the information provided. The median age at baseline (32 years) was used to create a dichotomous variable which was used in the analyses.

### Statistical analyses

Random effects linear regression models estimated using maximum likelihood, taking into account the cluster-based recruitment of the participants within parent groups, were used to assess the differences in outcomes between the intervention and control group. Due to the fact that some of the outcomes had highly skewed distributions, in all regression models se were bootstrapped (2000 re-samples). Models with BMI *z*-score as the outcome also included the child’s BMI *z*-score from baseline as a covariate. However, due to the fact that children were only 4 months of age at baseline, there are no baseline data for any of the dietary variables, television viewing or physical activity. Furthermore, for the models that included physical activity, ActiGraph wear time for that time point was used as a covariate.

In order to evaluate moderation by maternal education and age, models evaluating each outcome comprised terms for maternal education or age and intervention group (i.e., intervention or control group) as well as an interaction term (i.e., maternal education or age × intervention group). The intervention’s effect on the outcome was deemed to be moderated when the interaction term’s coefficient was significantly different to zero^(19)^. As moderator analysis in randomised controlled trial is used for both hypothesis formation and testing^(20)^ and due to the fact that testing for moderation is affected by the sample distribution and size^(21)^, a *P*-value of 0·2 was used to indicate significance in the moderation analyses. After the moderation analysis, the direction and strength of the intervention effect were investigated using a linear combinations post-estimation command to obtain the estimated intervention effect for the non-reference level of the moderator. All analyses were conducted with Stata (Release 15, StataCorp LP).

## Results

Table 1 presents the baseline characteristics of both the participating mother and child. At the 2- and 3·5-year follow-ups, no significant difference was observed for maternal education between the control group and intervention group (*χ* ^2^ = 2·5, *P* = 0·115 and *χ* ^2^ = 1·9, *P* = 0·173, respectively). No significant differences were observed for the dichotomised variables for maternal age between the intervention and control groups at either the 2-year (*χ* ^2^ = 0·1, *P* = 0·816) or 3·5-year follow-ups (*χ* ^2^ =0·1, *P* = 0·699).

**Table 1:** Baseline characteristics of the mothers and infants in INFANT

	2-year follow-up				3·5-year follow-up			
	Intervention (*n* 177)*		Control (*n* 181)		Intervention (*n* 185) *		Control (*n* 177)	
	Mean	sd	Mean	sd	Mean	sd	Mean	sd
Infants								
Age (months)	4·0	1·5	3·7	1·3	4·0	1·5	3·7	1·3
zBMI	−0·50	1·09	−0·58	0·98	−0·46	1·08	−0·58	0·99
Male								
*n*	92		99		98		93	
%	52·0		54·7		53·0		52·5	
Mothers								
Age (years)	32·6	4·3	32·4	4·3	32·7	4·1	32·4	4·3
Pre-pregnancy BMI (kg/m^2^)	24·4	5·6	23·8	4·5	24·3	5·5	24·3	5·2
Education level								
Low (all other)								
*n*	78		65		82		66	
%	44·1		35·9		44·3		37·3	
High (completed university degree or beyond)								
*n*	99		116		103		111	
%	55·9		64·1		55·7		62·7	

* One mother was missing pre-pregnancy BMI and therefore *n* 176 and 184 at the 2- and 3·5-year follow-ups, respectively.

### Moderating effect of maternal education at the 2- and 3·5-year follow-ups

Table 2 shows the moderation effect of maternal education on the intervention effect at the 2-year follow-up. It was observed that maternal education moderated the intervention effect for the consumption of vegetables (interaction, *P* = 0·090), water (interaction, *P* = 0·076) and sweet snacks (interaction, *P* = 0·079). Vegetable consumption was 30·26 g/d (95 % CI 11·03, 49·50) higher in children in the intervention group with university educated mothers compared to the control group with university educated mothers. The consumption of sweet snacks was –9·30 g/d (95 % CI –14·82, –3·79) lower in children in the intervention group with university educated mothers. Additionally, water intake was 184·63 g/d (95 % CI 47·31, 321·94) higher in children in the intervention group with non-university educated mothers.

**Table 2:** Assessment of the effect of the intervention in INFANT according to maternal education level at the 2-year follow-up*

	Effects of the intervention									
	Total			Education = low†			Education = high†			
	MD‡ beta§	95 % CI	*P*	MD‡ beta§	95 % CI	*P*	MD‡ beta§	95 % CI	*P*	Interaction (*P*)
zBMI	0·02	−0·11, 0·16	0·719	−0·02	−0·26, 0·23	0·906	0·05	−0·12, 0·22	0·587	0·704
Fruit intake (g/d)	24·59	0·74, 48·44	0·043	36·56	−16·40, 89·52	0·176	17·90	−12·51, 48·30	0·249	0·474
Vegetable intake (g/d)	16·34	−0·92, 33·60	0·064	0·96	−28·60, 30·52	0·949	30·26	11·03, 49·50	0·002	0·090
Water intake (g/d)	102·61	36·25, 168·97	0·002	184·63	47·31, 321·94	0·008	38·85	−31·44, 109·13	0·279	0·076
Non-core drinks intake (g/d)	12·45	−15·14, 40·04	0·376	21·75	−27·16, 70·65	0·383	3·21	−40·32, 46·75	0·885	0·614
Sweet snacks intake (g/d)	−5·87	−10·14, –1·61	0·007	−0·60	−8·20, 7·00	0·877	−9·30	−14·82, –3·79	0·001	0·079
Savoury snacks intake (g/d)	−0·51	−2·92, 1·89	0·676	−0·98	−6·66, 4·70	0·735	−0·33	−3·69, 3·02	0·845	0·864
Television viewing (min/d)	−5·01	−25·92, 15·90	0·638	−3·69	−51·15, 43·77	0·879	−15·63	−33·73, 2·47	0·091	0·665
Physical activity (min/d)||	1·29	−8·95, 11·53	0·805	19·94	−13·56, 53·45	0·243	−6·99	−23·95, 9·97	0·419	0·234

MD, mean difference

* Sample sizes: zBMI: total (*n* 354), low (*n* 143), high (*n* 211); dietary variables: total (*n* 261), low (*n* 98), high (*n* 163); television viewing: total (*n* 343), low (*n* 135), high (*n* 208); physical activity: total (*n* 144), low (*n* 44), high (*n* 100).

† High education = university degree; low education = all other.

‡ Mean difference coefficients between the intervention and control group estimated from linear regression analysis.

§ Random effects linear regression models, estimated using maximum likelihood with bootstrapped se, were fitted to compare continuous outcomes between the intervention and control groups. For the models that were measuring the intervention effects on zBMI and physical activity, they were adjusted for baseline zBMI and accelerometer wear time, respectively.

|| Total physical activity (i.e., includes light, moderate and vigorous intensity).

Moderation of the intervention effect by maternal education at the 3·5-year follow-up was observed for zBMI (interaction, *P* = 0·126) and water intake (interaction, *P* < 0·001) (Table 3). In stratified analyses, no significant differences were observed between mothers with and without a university education for zBMI or water intake. However, in line with the 2-year follow-up there was a trend for water intake to be greater in children in the intervention group with a non-university educated mothers (MD beta = +114·28 g/d, 95 % CI –38·75, 267·31).

**Table 3:** Assessment of the effect of the intervention in INFANT according to maternal education level at the 3·5-year follow-up*

	Effects of the intervention									
	Total			Education = low†			Education = high†			
	MD‡ beta§	95 % CI	*P*	MD‡ beta§	95 % CI	*P*	MD‡ beta§	95 % CI	*P*	Interaction (*P*)
zBMI	0·00	−0·19, 0·19	0·991	0·16	−0·06, 0·38	0·167	−0·11	−0·39, 1·17	0·434	0·126
Fruit intake (g/d)	2·00	−23·99, 28·00	0·880	12·57	−24·85, 49·99	0·510	−1·57	−37·20, 34·06	0·931	0·595
Vegetable intake (g/d)	5·87	−12·42, 24·17	0·529	4·71	−25·82, 35·24	0·762	9·98	−14·07, 34·03	0·416	0·798
Water intake (g/d)	26·81	−28·36, 81·99	0·341	114·28	−38·75, 267·31	0·143	−29·21	−109·47, 51·05	0·476	< 0·001
Non-core drinks intake (g/d)	−36·95	−62·31, –11·59	0·004	−43·60	−91·44, 4·24	0·074	−34·96	−70·49, 0·57	0·054	0·792
Sweet snacks intake (g/d)	−6·45	−11·63, –1·27	0·015	−7·75	−16·98, 1·47	0·099	−5·41	−13·39, 2·58	0·184	0·733
Savoury snacks intake (g/d)	−0·27	−3·25, 2·70	0·856	0·17	−6·10, 6·44	0·959	−1·44	−4·87, 1·99	0·410	0·669
Television viewing (min/d)	−8·31	−21·63, 5·01	0·221	−20·91	−48·86, 7·03	0·142	−2·51	−18·39, 13·36	0·756	0·294
Physical activity (min/d)||	1·97	−9·69, 13·63	0·741	7·47	−26·28, 41·21	0·664	−3·18	−19·03, 12·68	0·694	0·613

MD, mean difference

* Sample sizes: zBMI: total (*n* 362), low (*n* 148), high (*n* 214); dietary variables: total (*n* 270), low (*n* 103), high (*n* 167); television viewing: total (*n* 352), low (*n* 142), high (*n* 210); physical activity: total (*n* 146), low (*n* 47), high (*n* 99).

‡ Mean difference coefficients between the intervention and control group estimated from linear regression analysis.

† High education = university degree; low education = all other.

§ Random effects linear regression models, estimated using maximum likelihood with bootstrapped se, were fitted to compare continuous outcomes between the intervention and control groups. For the models that were measuring the intervention effects on zBMI and physical activity, they were adjusted for baseline zBMI and accelerometer wear time, respectively.

|| Total physical activity (i.e., includes light, moderate and vigorous intensity).

### Moderating effect of maternal age at the 2- and 3·5-year follow-ups

Table 4 shows the moderation effect of maternal age on the intervention effect at the 2-year follow-up. It was observed that maternal age moderated the intervention effect for water and sweet snack intake (interaction, *P* = 0·052 and 0·075, respectively) as well as for physical activity (interaction, *P* = 0·110). Water consumption was 177·36 g/d (95 % CI 80·54, 274·19) higher in children in the intervention group with mothers aged < 32 years compared to the control group with mothers aged < 32 years. Sweet snack consumption was −9·91 g/d (95 % CI –15·65, –4·17) lower in children in the intervention group with mothers aged ≥ 32 years. No significant intervention effect for physical activity was observed in children of either younger or older mothers; however, there was a trend for children in the intervention group with younger mothers to decrease physical activity (MD beta = –12·95 min/d, 95 % CI –33·92, 8·02) and children in the intervention group with older mothers to increase their physical activity (MD beta = +11·57 min/d, 95 % CI –3·76, 26·91).

**Table 4:** Assessment of the effect of the intervention in INFANT according to maternal age at the 2-year follow-up*

	Effects of the intervention									
	Total			Age < 32 years			Age ≥ 32 years			
	MD† beta‡	95 % CI	*P*	MD† beta‡	95 % CI	*P*	MD† beta‡	95 % CI	*P*	Interaction (*P*)
zBMI	0·02	−0·11, 0·16	0·719	0·07	−0·14, 0·27	0·508	−0·01	−0·22, 0·20	0·906	0·611
Fruit intake (g/d)	24·59	0·74, 48·44	0·043	23·28	−13·06, 59·62	0·209	22·98	−11·25, 57·21	0·188	0·991
Vegetable intake (g/d)	16·34	−0·92, 33·60	0·064	27·58	−1·82, 56·98	0·066	6·97	−14·85, 28·80	0·531	0·287
Water intake (g/d)	102·61	36·25, 168·97	0·002	177·36	80·54, 274·19	<0·001	42·34	−52·38, 137·06	0·381	0·052
Non-core drinks intake (g/d)	12·45	−15·14, 40·04	0·376	5·54	−46·08, 57·16	0·833	19·87	−15·00, 54·75	0·264	0·672
Sweet snacks intake (g/d)	−5·87	−10·14, –1·61	0·007	−1·09	−8·21, 6·04	0·765	−9·91	−15·65, –4·17	0·001	0·075
Savoury snacks intake (g/d)	−0·51	−2·92, 1·89	0·676	−0·44	−4·94, 4·06	0·849	−0·51	−4·82, 3·79	0·816	0·984
Television viewing (min/d)	−5·01	−25·92, 15·90	0·638	5·80	−25·21, 36·82	0·714	−14·04	−43·79, 15·71	0·355	0·380
Physical activity (min/d)§	1·29	−8·95, 11·53	0·805	−12·95	−33·92, 8·02	0·226	11·57	−3·76, 26·91	0·139	0·110

MD, mean difference

* Sample sizes: zBMI: total (*n* 354), age < 32 years (*n* 161), age ≥ 32 years (*n* 193); dietary variables: total (*n* 261), < 32 years (*n* 119), age ≥ 32 years (*n* 142); television viewing: total (*n* 343), age < 32 years (*n* 156), age ≥ 32 years (*n* 187); physical activity: total (*n* 144), age < 32 years (*n* 59), age ≥ 32 years (*n* 85).

† Mean difference coefficients between the intervention and control group estimated from linear regression analysis.

‡ Random effects linear regression models, estimated using maximum likelihood with bootstrapped se, were fitted to compare continuous outcomes between the intervention and control groups. For the models that were measuring the intervention effects on zBMI and physical activity, they were adjusted for baseline zBMI and accelerometer wear time, respectively.

§ Total physical activity (i.e., includes light, moderate and vigorous intensity).

Moderation of the intervention effect by maternal age at the 3·5-year follow-up was observed for the intake of fruit (interaction, *P* = 0·046), sweet snacks (interaction, *P* = 0·136), savoury snacks (interaction, *P* = 0·003) as well as physical activity (interaction, *P* = 0·200) (Table 5). Sweet snack intake was –11·54 g/d (95 % CI –20·57, –2·52) lower in children in the intervention group with mothers aged < 32 years compared to the control group with mothers aged < 32 years. With regard to savoury snacks, significant intervention effects in both mothers aged < 32 years and ≥ 32 years were observed in stratified analyses. Children in the intervention group with younger mothers significantly increased their consumption of savoury snacks by 5·56 g/d (95 % CI 0·70, 10·41), whereas children in the intervention group with older mother significantly decreased their consumption of savoury snacks by –5·15 g/d (95 % CI –9·48, –0·81). No significant intervention effect for fruit intake or physical activity was observed in children of either younger or older mothers. However, there was a trend for children in the intervention group with younger mothers to increase their fruit intake (MD beta = +38·40 g/d, 95 % CI –3·29, 80·10) and children in the intervention group with older mothers to decrease their fruit intake (MD beta = −29·37 g/d, 95 % CI –72·19, 13·44). Similar to the 2-year follow-up, there was also a trend for children in the intervention group with younger mothers to decrease their physical activity (MD beta = –9·38 min/d,95 % CI –32·77, 14·00) and children in the intervention group with older mothers to increase their physical activity (MD beta = +10·42 min/d, 95 % CI –4·80, 25·63).

**Table 5:** Assessment of the effect of the intervention in INFANT according to maternal age at the 3·5-year follow-up*

	Effects of the intervention									
	Total			Age < 32 years			Age ≥ 32 years			
	MD† beta‡	95 % CI	*P*	MD† beta‡	95 % CI	*P*	MD† beta‡	95 % CI	*P*	Interaction (*P*)
zBMI	0·00	−0·24, 0·24	0·993	−0·05	−0·31, 0·21	0·709	0·04	−0·18, 0·26	0·700	0·435
Fruit intake (g/d)	2·00	−23·56, 27·57	0·878	38·40	−3·29, 80·10	0·071	−29·37	−72·19, 13·44	0·179	0·046
Vegetable intake (g/d)	5·87	−12·91, 24·66	0·540	4·70	−19·42, 28·82	0·703	6·67	−18·38, 31·72	0·602	0·905
Water intake (g/d)	26·81	−22·79, 76·42	0·289	64·14	−44·00, 172·25	0·245	−3·61	−126·42, 119·19	0·954	0·479
Non-core drinks intake (g/d)	−36·95	−62·76, –11·12	0·005	−39·17	−88·29, 9·94	0·118	−34·80	−67·45, –2·14	0·037	0·892
Sweet snacks intake (g/d)	−6·45	−11·82, –1·07	0·019	−11·54	−20·57, –2·52	0·012	−2·11	−9·38, 5·16	0·569	0·136
Savoury snacks intake (g/d)	−0·27	−3·40, 2·85	0·863	5·56	0·70, 10·41	0·025	−5·15	−9·48, –0·81	0·020	0·003
Television viewing (min/d)	−8·31	−21·82, 5·20	0·228	−10·34	−25·82, 5·13	0·190	−7·01	−28·85, 14·83	0·529	0·810
Physical activity (min/d)§	1·97	−9·59, 13·53	0·738	−9·38	−32·77, 14·00	0·432	10·42	−4·80, 25·63	0·180	0·200

MD, mean difference

* Sample sizes: zBMI: total (*n* 362), < 32 years (*n* 166), ≥ 32 years (*n* 196); dietary variables: total (*n* 270), < 32 years (*n* 123), ≥ 32 years (*n* 147); television viewing: total (*n* 352), < 32 years (*n* 163), ≥ 32 years (*n* 189); physical activity: total (*n* 146), < 32 years (*n* 64), ≥ 32 years (*n* 82).

† Mean difference coefficients between the intervention and control group estimated from linear regression analysis.

‡ Random effects linear regression models, estimated using maximum likelihood with bootstrapped se, were fitted to compare continuous outcomes between the intervention and control groups. For the models that were measuring the intervention effects on zBMI and physical activity, they were adjusted for baseline zBMI and accelerometer wear time, respectively.

§ Total physical activity (i.e., includes light, moderate and vigorous intensity).

## Discussion

The current study examined how maternal education and age moderate the effect of INFANT 2 and 3·5 years post-intervention. At both follow-ups, maternal education and age moderated some of the intervention’s effects; however, clear patterns were not evident at the long-term follow-ups. At the 2-year follow-up, the results regarding maternal education were similar to those obtained post-intervention^(10)^. However, these results were not sustained at the 3·5-year follow-up. With regard to maternal age, inconsistent results were obtained across post-intervention^(10)^ and both follow-ups. These inconsistent results and the fact that many of the outcomes were not moderated by maternal age or education indicate that INFANT was more-or-less equally effective in children irrespective of their mother’s education level or age.

### Maternal education

Similar to what was observed at the end of INFANT^(10)^, at the 2-year follow-up children in the intervention group with university educated mothers consumed significantly more vegetables and less sweet snacks compared to their peers whose mother’s had no university education. However, these effects were not sustained at the 3·5-year follow-up. A recent systematic review by Hodder *et al.* ^(22)^ found moderate-quality evidence for multi-component interventions to increase vegetable consumption in children under 5 years which included parental involvement in the intervention, a focus on vegetable consumption instead of general nutrition knowledge and nutrition education. The focus on specific messaging and skill development around vegetables and other core and non-core foods that were the focus of INFANT may have supported the vegetable differences observed early in life, but appears to have been more effective for university educated mothers in a child’s early years. The 30 g/d increase in vegetables that was observed in children with university educated mothers at the 2-year follow-up equals approximately half a serving (defined as 75 g), which is about one fifth of the daily serving for a 2-3-year-old child^(23)^. Furthermore, in cross-sectional and longitudinal studies, healthier dietary patterns have also been observed in infants/young children whose mothers have a higher education^(9,24)^, suggesting this dietary change may be easier to achieve in higher educated groups. Future research should investigate if interventions need to be sustained in order to maintain effects on vegetable consumption.

To the best of our knowledge, no other obesity prevention interventions to date have investigated maternal education as a moderator in long-term follow-ups. However, in addition to INFANT^(10)^, two other studies have investigated potential moderation at the end of their interventions. At the intervention conclusion in the Healthy School Start II and ToyBox interventions, no moderation for any of the dietary outcomes or diet quality by parental or maternal education was observed^(25,26)^. The differing results obtained by the current study and these studies could be due to the fact that Healthy School Start II and ToyBox were based in the school and kindergarten setting, whereas INFANT was community based and focused on parents as the agents of change. Furthermore, differences could be attributed to the different age of the children when the interventions were delivered, that is, infants in the current study, 6 years of age in a Healthy School Start II^(25)^ and between 3·5 and 5·5 years in the ToyBox study^(26)^. Interventions starting in infancy may be more effective than interventions starting later in life as it has been found that parents are most responsive during infancy^(27)^. Finally, the differences could also be due to how the dietary data were collected in the studies, with 24-h recalls used in INFANT and FFQ used in the other two studies^(25,26)^. This might have influenced the observed differences between the studies as dietary recalls are a more precise method than FFQ.

In contrast to the other dietary variables, and as observed at the end of INFANT^(10)^, a significant increase in water consumption was observed among children with non-university educated mothers. The significant difference was only observed at the 2-year follow-up; however, there was a trend showing that children of non-university educated mothers consumed more water than children with university educated mothers at the 3·5-year follow-up (+114·28 g/d, *P* = 0·143 *v*. –29·21 g/d, *P* = 0·476). In a cross-sectional analysis using the baseline data from ToyBox, children with lower educated mothers consumed significantly less water than children with higher educated mothers in the total sample^(28)^. As the results of INFANT indicate that children with non-university educated mothers consumed more water, this could indicate that water intake is a behaviour that can be successfully changed in interventions in lower socio-economic groups. However, this could differ between countries depending on tap water quality and if tap water is perceived as safe. In Australia, tap water is free and accessible and there has been nationwide campaigns from the government (e.g., ‘Choose TAP’^(29)^) to promote the consumption of tap water. In the European Union, bottled water consumption is prevalent and tap water is overlooked due to perceived notions regarding quality^(30)^. Thus, increasing water consumption in lower socio-economic groups may be more effective in countries such as Australia where tap water is perceived as safe, and its minimal cost becomes a facilitator of change particularly to lower socio-economic groups.

### Maternal age

At the 2-year follow-up, children in the intervention group with younger mothers consumed significantly more water than their counterparts with older mothers. This result was also observed post-intervention^(10)^; however, it was not sustained at the 3·5-year follow-up. In a recent systematic review by Franse *et al.* ^(31)^, maternal age was not found to be a factor associated with water consumption in children. The results observed in the present study could be due to the fact that younger mothers may be more receptive to change as they may not have as strongly developed views regarding parenting-related behaviours as older mothers.

At the 2-year follow-up, sweet snack consumption significantly decreased in children with older mothers. However, at the 3·5-year follow-up the opposite result was found, where sweet snack consumption decreased in children with younger mothers. Furthermore, at the 3·5-year follow-up it was found that the children’s consumption of savoury snacks significantly increased in those with younger mothers and significantly decreased in those with older mothers. While it is unclear why these results were observed, previous studies have found that greater maternal age (≥ 30 years) has been characterised by healthier dietary patterns in infants^(9)^. Thus, at the 3·5-year follow-up it could be hypothesised that the younger mothers substituted sweet snacks for savoury snacks and the children’s overall intake of unhealthy snacks did not decrease. However, there was also a trend showing that the intake of fruit in children with younger mothers increased; therefore, sweet snacks could have also been replaced with fruit at this time point.

### Strengths and limitations

Strengths of this study include the long-term follow-ups, use of multi-pass 24- h dietary recalls, objective measures of physical activity as well as reliable measure for television viewing. Furthermore, this study reports the moderation analyses of the intervention effects at two long-term follow-ups, which is currently lacking in the contemporary literature regarding obesity prevention interventions in young children. It is important to note that the mother infant dyads excluded from this study at the 2- and 3·5-year follow-ups because of missing data had lower education than the included sample. Another limitation is that change in maternal education level as well as attendance at child care was not accounted for in this study. Additionally, as INFANT was not powered based on assessment of the moderation analyses, the sample size may have been too small to detect some true differences where they actually exist. Larger studies that would permit detection of intervention effects in subgroups, such as maternal education and age, are warranted.

## Conclusions

Moderation by maternal education and age were observed for some outcomes; however, clear patterns were not evident at both follow-ups, with little consistency across outcomes. This indicates that INFANT was more-or-less equally effective in children irrespective of their mother’s education level or age, which is important in community-based interventions.
